# Hepatitis C prevalence in incarcerated settings between 2013–2021: a systematic review and meta-analysis

**DOI:** 10.1186/s12889-022-14623-6

**Published:** 2022-11-24

**Authors:** Dana Busschots, Cécile Kremer, Rob Bielen, Özgür M. Koc, Leen Heyens, Frederik Nevens, Niel Hens, Geert Robaeys

**Affiliations:** 1 Faculty of Medicine and Life Sciences, Hasselt University, Martelarenlaan 42, Diepenbeek, 3500 Hasselt, Belgium; 2 Department of Gastroenterology and Hepatology, Ziekenhuis Oost-Limburg, Genk, Belgium; 3 Interuniversity Institute for Biostatistics and Statistical Bioinformatics (I-Biostat), Data Science Institute, Hasselt University, Diepenbeek, Belgium; 4 Medical Microbiology, School of NUTRIM, Maastricht University Medical Centre, Maastricht, the Netherlands; 5 School of NUTRIM, Maastricht University Medical Centre, Maastricht, the Netherlands; 6 Department of Gastroenterology and Hepatology, University Hospitals KU, Leuven, Belgium; 7 Centre for Health Economic Research and Modelling Infectious Diseases, Vaccine and Infectious Disease Institute, University of Antwerp, Antwerp, Belgium

**Keywords:** Hepatitis c, Incarcerated setting, Prevalence, Global health, Meta-analysis

## Abstract

**Background:**

The introduction of highly effective direct-acting antiviral therapy has changed the hepatitis C virus (HCV) treatment paradigm. However, a recent update on HCV epidemiology in incarcerated settings is necessary to accurately determine the extent of the problem, provide information to policymakers and public healthcare, and meet the World Health Organization's goals by 2030. This systematic review and meta-analysis were performed to determine the prevalence of HCV Ab and RNA in incarcerated settings.

**Methods:**

For this systematic review and meta-analysis, we searched PubMed, Embase, Scopus, and Web of Science for papers published between January 2013 and August 2021. We included studies with information on the prevalence of HCV Ab or RNA in incarcerated settings. A random-effects meta-analysis was done to calculate the pooled prevalence and meta-regression to explore heterogeneity.

**Results:**

Ninety-two unique sources reporting data for 36 countries were included. The estimated prevalence of HCV Ab ranged from 0.3% to 74.4%. HCV RNA prevalence (available in 46 sources) ranged from 0% to 56.3%. Genotypes (available in 19 sources) 1(a) and 3 were most frequently reported in incarcerated settings. HCV/HIV coinfection (available in 36 sources) was highest in Italy, Estonia, Pakistan, and Spain. Statistical analysis revealed that almost all observed heterogeneity reflects real differences in prevalence between studies, considering I^2^ was very high in the meta-analysis.

**Conclusions:**

HCV in incarcerated settings is still a significant problem with a higher prevalence than in the general population. It is of utmost importance to start screening for HCV (Ab and RNA) in incarcerated settings to give clear, reliable and recent figures to plan further treatment. This is all in the context of meeting the 2030 WHO targets which are only less than a decade away.

**Trial registration:**

PROSPERO: CRD42020162616

**Supplementary Information:**

The online version contains supplementary material available at 10.1186/s12889-022-14623-6.

## Background

Chronic hepatitis C virus (HCV) infection remains a global health problem prompting the World Health Organization (WHO) to define elimination goals by 2030 (by reducing new infections by 90% and mortality by 65%) [1]. One of the key risk groups for HCV infection is people with a history of (injecting) drug use, with the majority having an opioid use disorder [2, 3]. These individuals often have a lower social health status (e.g., low education level, unstable housing) compared to the general population and are often marginalized [4]. Subsequently, they are difficult to reach and have reduced access to healthcare services [5]. Since drug use is illegal in most countries, these individuals are more likely to end up in incarcerated settings. Prior studies indicate that individuals who are incarcerated are more likely to engage in HCV-related risk behavior such as unsterile tattooing, high-risk sexual behavior, and sharing paraphernalia [2, 6]. Worldwide, this has led to an increased prevalence of HCV in individuals who are incarcerated compared to the general population. The prevalence of HCV antibodies (Ab) in closed settings was estimated in 2013 by Larney et al. to be 30% in Western Europe and varied worldwide with prevalences up to 35% in Australia and 38% in Central Asia [6, 7]. In 2016, Dolan et al. showed a decrease in the estimated pooled HCV Ab prevalence in Western European incarcerated settings (15.5%) [6]. In contrast, HCV Ab is present in 2–3% of the global population [8]. Given that at any moment, an estimated 1.6 million people are incarcerated in Europe and even 10.7 million worldwide, individuals who are incarcerated are a key group to combat for HCV elimination [9].

The high occurrence of HCV infection in individuals who are incarcerated and the substantial risks associated with untreated HCV infection underline the need for HCV screening and access to treatment in individuals who are incarcerated [10]. The WHO, therefore, recommends that all individuals who are incarcerated should be tested for HCV at least once during their stay [11]. However, adherence to WHO guidelines for HCV screening in incarcerated settings is currently inadequate. In Europe, only ten (34.4%) of 29 surveyed countries reported HCV screening programs for individuals who are incarcerated in 2010 [10, 12]. Since then, only scarce data on HCV prevalence in individuals who are incarcerated have been published in Europe or other regions [13]. Also, chronic HCV infection leads to cirrhosis and potentially hepatocellular carcinoma if untreated and is one of the leading causes of liver transplantations in high-income countries [14]. However, neither Larney et al. nor Dolan et al. reported data on HCV RNA or HCV genotype. Nevertheless, they suggested that HCV screening and subsequent antiviral treatment in this group of patients is necessary and will reduce the global HCV transmission rate [15, 16].

To determine the extent of the problem and provide guidance to policymakers and public healthcare, a better understanding of the epidemiology of HCV infection in individuals who are incarcerated is necessary. A valuable meta-analysis on HCV prevalence in closed settings worldwide was published in 2013. This review showed high heterogeneity among prevalence estimates from the different data sources [7]. To reach the WHO goals by 2030, we need to systematically map this global health problem in all risk groups, including individuals who are incarcerated.

Elimination of HCV (reducing new infections by 90% and 80% of eligible patients being treated), even in incarcerated settings, is possible as the Trap HepC study from Iceland demonstrates. They offered screening and (simultaneous) treatment for HCV to all individuals who were incarcerated. If released during treatment, the individual who was incarcerated was followed at one of the TraP HepC treatment sites to facilitate the elimination of HCV in formerly incarcerated persons in the community [17].

Therefore, we performed an update and a new systematic review and meta-analysis on HCV in incarcerated settings worldwide. This review will report not only data on HCV Ab but, for the first time, also on HCV RNA and genotype in incarcerated settings. HCV Ab prevalence will shed light on the overall problem and the need for testing and monitoring. HCV RNA prevalence indicates the necessity of antiviral treatment. Genotyping is essential for countries that do not have access to pangenotypic antivirals. Since HIV is a risk factor for HCV, we also aim to assess the extent to which HCV/HIV coinfection is present in incarcerated settings worldwide.

## Materials and methods

### Search strategy

This systematic review and meta-analysis is reported in line with the PRISMA checklist [see Additional file A1] [18] and registered at PROSPERO (CRD42020162616). To assess HCV prevalence in individuals who are incarcerated worldwide, a structured approach was followed. For this purpose, PubMed, Embase, Scopus, and Web of Science databases were used.

The following terms and keywords alone and/or in appropriate combinations were included in the search: “hepatitis C,” “HCV,” “antibodies,” “RNA,” “jail,” “prevalence,” and “prison.” The search was limited to full articles or abstracts in the English language and published between January 2013 and August 2021 [see Additional file A2]. This date limit was set as in 2013, Larney et al*.* published an essential meta-analysis on HCV prevalence in closed settings worldwide [7].

All search results were systematically screened by the first author and last author and documented using EndNote X8. The snowball method was used to enrich the results, meaning that reference lists of included articles were screened for relevant articles.

### Data extraction

A quality assessment of the found sources was carried out. To grade and select studies for inclusion, available methodological information was used, and the principles defined in Nelson et al*.* were used to grade the data [19]. Ungraded studies (i.e., unknown methodology) were excluded [see Additional file A3].

We selected HCV prevalence studies in a incarcerated setting (prison, jail, or pre-trial detention center). Studies using self-reported HCV status, saliva, dried blood spot sampling or RNA testing only were also included though they were graded lower [see Additional file A3]. Participants in the included studies were defined as individuals who are incarcerated at a criminal justice facility with any sentence duration and aged 18 years or older. We explicitly excluded studies with a study population consisting of individuals who are incarcerated not by criminal justice (war prisoners, concentration camps, persons in police custody, and persons in migrant centers), individuals with a history of incarceration or ex-convicts, and individuals who are incarcerated aged 17 years or younger. Publication types such as guidelines, perspectives, correspondence, systematic reviews or meta-analyses were excluded as well.

We extracted the following data from the articles included in this study: study sample, enrolment dates, country, number of subjects studied, number of persons positive for HCV Ab and/or HCV RNA, HCV genotypes, and HIV/HCV coinfection. If the article reported risk factors significantly associated with HCV prevalence, these were also extracted and are shown in [see Additional file A5].

### Statistical analyses

All statistical analyses were performed in R version 3.6.1. [20]. In this meta-analysis, studies in individuals who are incarcerated selected for the systematic review were included if they estimated HCV prevalence. If the exact number of cases was unknown, this was computed from prevalence and sample size and rounded upward. Sensitivity analysis was performed by rounding these numbers downward. Classic random-effects meta-analysis (i.e., inverse variance method) with double arcsine transformed proportions was used. Since the analysis results using double arcsine transformed proportions may be seriously misleading when the individual sample sizes vary (in this study ranging from 133 to 101,727), logit transformed proportions were used as a sensitivity analysis. A generalized linear mixed model (GLMM; random-intercept logistic regression model, with logit transformed proportions) was also used as recommended by Schwarzer et al*.* [21].

Heterogeneity was investigated using the I^2^ measure, which ranges from 0 to 100% and reflects the proportion of the observed variance that reflects true differences in prevalence estimates not attributable to chance [22]. To investigate possible sources of between-study heterogeneity, subgroup analyses based on region (Table 1), publication year, study size, type of study, single vs. multi-site were performed, and to whether people who inject drugs (PWID) or HIV-coinfected population were included. Next, a meta-regression analysis was performed. First, univariate models were used to examine which study characteristics could explain some of the between-study variability. Study characteristics included were region, publication year, sample size, study type, single- or multi-centric, the proportion of HIV coinfected individuals, the proportion of PWID, and the proportion of males. All characteristics with a *p*-value < 0.200 were then included in a multivariable meta-regression model, and backward selection was applied, removing all non-significant covariates in a stepwise manner.

**Table 1:** Results of a systematic review on hepatitis C virus prevalence in incarcerated settings

	Author	Publication year	Sample size	Enrolment dates	Prevalence HCV (%)	
					Ab	RNA
**Eastern Europe**						
Azerbaijan	Azbel L et al. [23]	2015	510	2014	38.2	-
Bosnia and Herzegovina	Hodzic H et al. [24]	2017	200	2013	13.0	-
Estonia	Kivimets K et al. [25]	2018	1,845	2014–2015	-	56.3
Georgia	Bergen-Cico D et al. [26]	2017	500	2016	60.0	-
	Harris AM et al. [27]	2019	13,500	2013–2015	38.0	28.4
Hungary	Vanya M et al. [28]	2017	200	2014	1.0	-
Macedonia	Jovanovska T et al. [29]	2014	200	-	20.0	-
Turkey	Keten D et al. [30]	2016	266	2014–2015	17.7	8.6
	Kose S et al. [31]	2019	360	-	0.5	-
Ukraine	Azbel L et al. [32]	2013	402	2011	60.2	-
**Western Europe**						
Austria	Silbernagl M et al. [33]	2018	133	-	74.4	45.0
Belgium	Busschots D et al. [34]	2021	886	2019–2020	5.0	2.1
Denmark	Soholm J et al. [35]	2019	801	2016–2017	7.4	4.2
France	Semaille C et al. [36]	2013	2,154	2010	4.8	2.5
	Roux P et al. [37]	2014	5,957	2004–2010	5.2	-
	Jacomet C et al. [38]	2016	342	2012–2013	4.7	1.5
	Izquierdo L et al. [39]	2019	1,093	2017	2.9	1.1
Ireland	Crowley D et al. [40]	2019	422	2017	22.8	5.5
Italy	Brandolini M et al. [41]	2013	695	2006–2008	22.4	19.4
	Foschi A et al. [42]	2016	3,400	-	10.0	6.0
	Masarone M et al. [43]	2020	458	2018–2019	12.7	10.0
	Fiore V et al. [44]	2021	2,376	2019	10.4	4.3
Spain	Marco, A et al. [45]	2014	2,377	1992–2011	-	4.9
	Cuadrado A et al. [46]	2018	847	2016–2017	13.0	10.2
	Jiménez Galan G et al. [47]	2019	1,200	2015	12.4	-
	Cabezas J et al. [48]	2021	548	2019–2021	8.0	2.9
United Kingdom	Taylor A et al. [49]	2013	4,904	2010–2011	19.0	-
	Aisyah D et al. [50]	2018	511	2011–2013	4.0	3.1
	Morey S et al. [51]	2019	1,495	2016–2017	6.4	3.1
	Perrett S et al. [52]	2019	256	2016	3.1	-
	Bhandari R et al. [53]	2020	8,538	2016–2020	7.2	4.4
	Mohamed Z et al. [54]	2020	2,442	2017–2019	3.7	2.6
	Perrett S et al. [55]	2020	6,949	2015–2017	11.0	-
	Gahrton C et al. [56]	2019	471	2017	17.0	11.5
Switzerland	Chacowry P et al. [57]	2018	273	2011–2013	6.2	-
**Northern America**						
Canada	Courtemanche Y et al. [58]	2018	1,315	2014–2015	12.9	-
United States	Alvarez KJ et al. [59]	2013	2,788	2009–2013	-	10.1
	Cocoros N et al. [60]	2014	596	2009–2011	20.5	-
	Wenger PJ et al. [61]	2014	304	2012–2013	16.4	-
	Kuncio D et al. [62]	2015	51,562	2011–2012	3.0	-
	Beckwith C et al. [63]	2016	249	2012–2014	9.2	6.0
	Mahowald M et al. [64]	2016	101,727	2004–2012	18.7	5.2
	Schoenbachler B et al. [65]	2016	893	2012–2014	13.2	7.4
	Stockman LJ et al. [66]	2016	1,239	2014–2015	12.5	8.9
	Akiyama M et al. [67]	2017	10,856	2013–2014	20.6	-
	de la Flor C et al. [68]	2017	3042	2015–2016	16.4	-
	Hochstatter K et al. [69]	2017	22,918	2015	-	13.6
	Assoumou SA et al. [70]	2019	24,567	2012–2016	20.0	7.0
	Abe C et al. [71]	2020	4,089	2017	17.3	10.1
	Chan J et al. [72]	2020	40,219	2014–2017	-	11.6
	Qureshi N et al. [73]	2021	80,681	2000–2019	34.6	-
**Latin America**						
Argentina	Adaszko D et al. [74]	2018	2,181	2015–2017	3.3	-
	Mendizabal M et al. [75]	2020	1,141	2018–2020	-	1.1
Brazil	Barros LA et al. [76]	2013	148	2007–2008	6.1	3.4
	Falquetto T et al. [77]	2013	730	2010	1.0	0.8
	El Maerrawi I et al. [78]	2015	680	2007	5.3	-
	Puga M et al. [79]	2017	3,368	2013–2014	2.4	1.5
	Felisberto M et al. [80]	2019	147	2015	5.4	-
	Machado F et al. [81]	2019	349	2016–2017	-	8.3
	Do Nascimento CT et al. [82]	2020	37,497	2017–2018	-	0.2
Mexico	Bautista-Arredondo S et al. [83]	2015	17,296	2010	3.2	-
	Belaunzaran-Zamudio P et al. [84]	2017	3,192	2011–2012	4.8	-
	Silverman-Retana O et al. [85]	2017	391	2010–2011	3.3	-
**Central Asia**						
Kyrgyzstan	Azbel L et al. [86]	2016	368	2014	42.4	-
**East Asia**						
Taiwan	Yang TH et al. [87]	2020	824	2019	33.5	23.2
**South Asia**						
India	Ramamoorthy M et al. [88]	2016	1,381	2015	1.2	-
	Tyagi SK et al. [89]	2018	1,611	2016	10.4	-
Indonesia	Prasetyo AA et al. [90]	2013	375	2009	34.1	
Pakistan	Pervaiz A et al. [91]	2015	5,894	2007–2009	-	14.6
	Wali A et al. [92]	2019	346	2017	10.4	-
Sri Lanka	Niriella MA et al. [93]	2015	393	-	6.9	0.5
**West Asia**						
Iran	Salem F et al. [94]	2013	3,000	2008–2009	0.7	-
	Ziaee M et al. [95]	2014	881	2009–2010	-	7.7
	Khajedaluee M et al. [96]	2016	1,114	2008	24.5	19.1
	Moradi G et al. [97]	2018	6,200	2015	9.5	
	Ghafari S et al. [98]	2019	300	2016	8.0	-
	Khademi N et al. [99]	2019	1,034	-	22.2	-
	Moradi G et al. [100]	2019	6,481	2016	8.2	-
	Sharafi H et al. [101]	2019	1,788	2017–2018	5.9	-
	Hariri S et al. [102]	2020	3,485	2017–2018	5.2	3.4
	Hariri S et al. [103]	2020	1,892	2018	6.9	4.8
**Australasia**						
Australia	Reekie JM et al. [104]	2014	1,393	2004–2007-2010	29.8	-
	Cunningham E et al. [105]	2017	320	2005–2014	29.1	-
	Snow K et al. [106]	2017	1,315	2008–2010	33.8	-
	Hajarizadeh B et al. [107]	2021	3?691	2018	-	19.0
	Sullivan RP et al. [108]	2021	12,153	2003–2017	4.7	-
**West Africa**						
Senegal	Jaquet A et al. [109]	2016	333	2014	0.6	-
Togo	Jaquet A et al. [109]	2016	347	2013	0.3	-
Nigeria	Okafor IM et al. [110]	2020	142	2018	29.6	-
**Sub Saharan Africa**						
Ethiopia	Wakjira K et al. [111]	2017	156	2016	2.6	-
	Kassa Y et al. [112]	2021	339	2020	1.2	-
**Middle East and North Africa**						
Egypt	Mohamed HI et al. [113]	2013	500	-	15.8	12.2

Prevalence HCV RNA is the prevalence relative to the total sample size

*Abbreviations*: *HCV* hepatitis c virus, *Ab* antibody

## Results

**Fig. 1:**
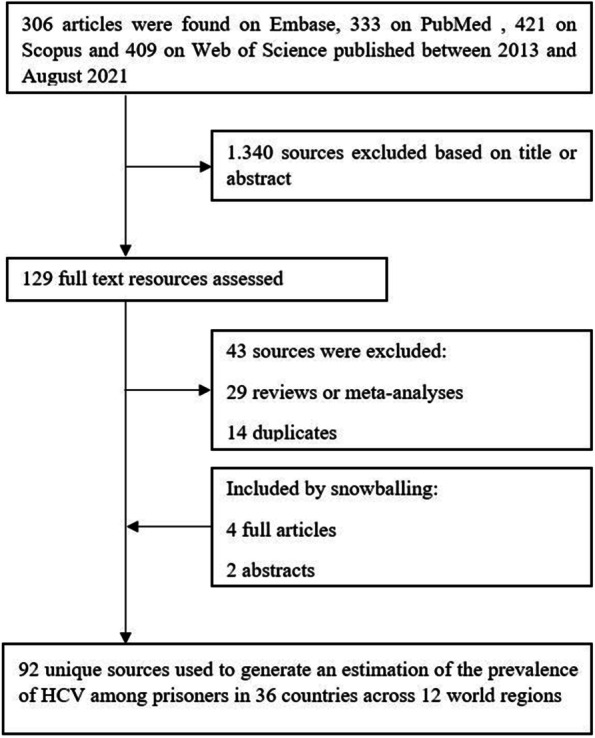
Search process and flowchart

The data search resulted in 306 potential data sources found on Embase, 333 on PubMed, 421 on Scopus, and 409 on Web of Science. The abstracts of these articles were systematically screened. Whereas 1,340 articles were excluded, 129 were thoroughly reviewed. While reviewing these articles, the snowball method was applied and another four articles and two abstracts were included. Twenty-nine articles were identified as reviews or meta-analyses, and 14 articles were duplicates and therefore excluded. This resulted in 90 eligible articles and two abstracts, reporting data for 36 different countries (Fig. 1) [see Additional file A4].

Results of the quality assessment showed that half of the articles were rated A, B1, or B2, respectively 4 (4.3%), 38 (41.3%) and 2 (2.2%). The other half was rated C, D or E, respectively 35 (38.0%), 7 (7.6%) and 6 (6.5%).

**Fig. 2:**
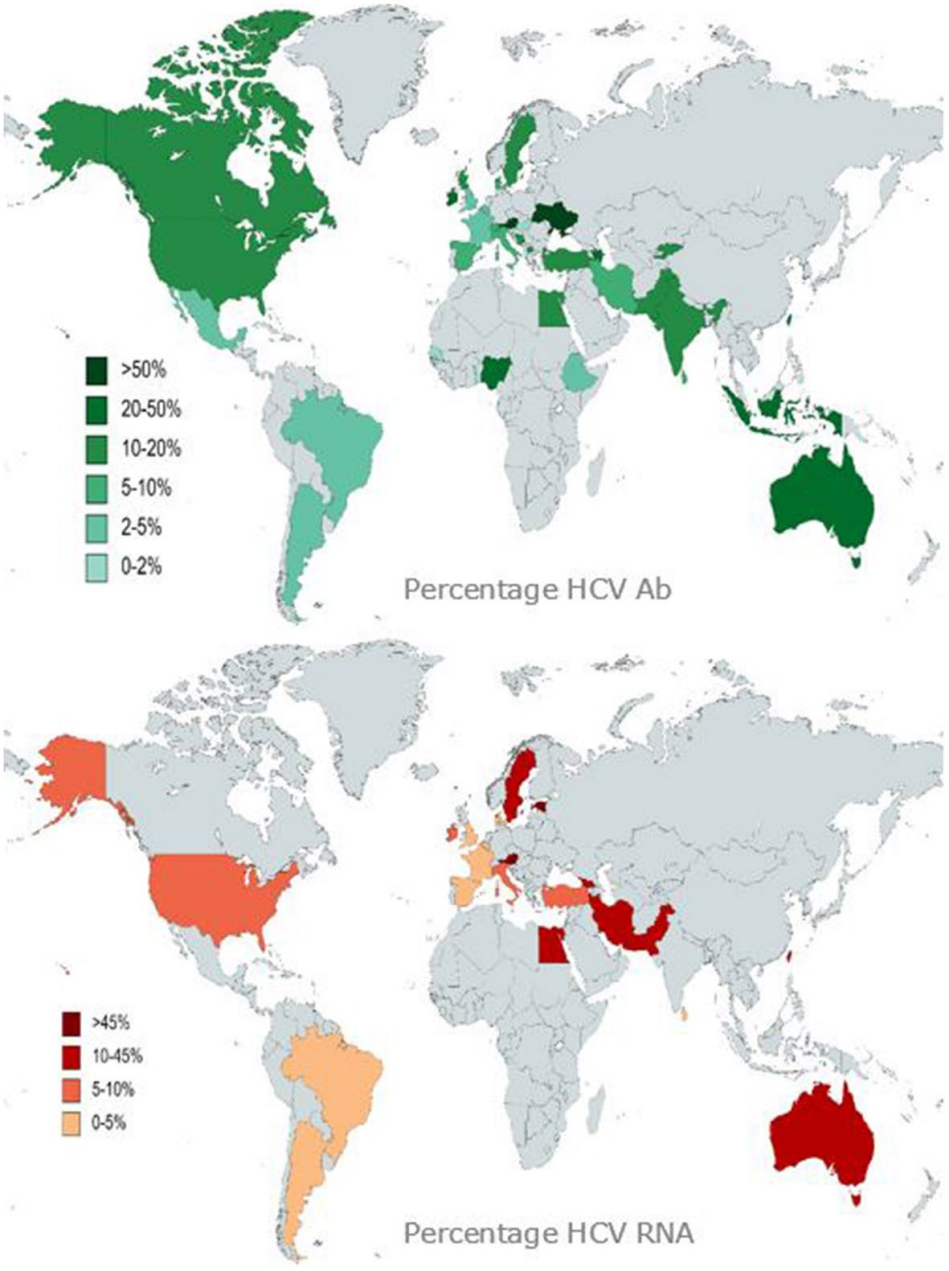
The global prevalence of hepatitis C in incarcerated settings Generated with data from the studies that were best graded as in [see Additional file A3]

The estimated prevalence of HCV Ab ranged from 0.3% to 74.4%. Jaquet et al*.* (Fig. 2) reported the lowest prevalence in 347 individuals who are incarcerated held in the state prison of Lome, Togo (Table 1) [109]. None of the HCV Ab positives individuals in Lome reported a history of intravenous drug use (IVDU) and information on other transmission routes was unavailable. The same authors found similar results for a prison in Dakar, Senegal (0.6%).

In contrast, the highest prevalence of HCV Ab was reported in Silbernagl et al*.* in the smallest study, with 133 individuals who are incarcerated receiving opioid agonist therapy (OAT) in four different prisons in Austria [33]. In this study, HCV Ab positivity was related to lifetime IVDU and younger age at first IVDU. The second highest prevalence (60.2%) was found in Ukraine in soon-to-released individuals who are incarcerated [32].

HCV RNA was available in 46/92 (50.0%) sources and ranged from 0% to 56.3% (Fig. 2). The lowest prevalence (0.2%) was reported by do Nascimento et al*.* in 37,497 individuals who are incarcerated in Brazil. Kivimets et al. reported the highest prevalence among 1,845 newly incarcerated individuals who are incarcerated in Estonia [25, 82]. Only 19 (41.3%) of these articles mentioned the HCV genotypes. The genotypes most frequently reported in incarcerated settings were 1(a) and 3 (Table 2).

**Table 2:** Distribution of genotypes in incarcerated settings (%)

COUNTRY	1	1A	1B	1C	2	2A/C	3	4	6
**Estonia**		17.0	35.2		2.6		44.4	0.8	
**Georgia**	22.7				28.8		47.7		
**Turkey**	2.1				68.0				
**Austria**	52.8				3.8	1.6	35.8	7.5	
**France**		50.0					41.7	8.3	
**Ireland**		58.7					41.3		
**Italy**	48.0				4.0		26.0	10.0	
		45.6	8.7		6.5		32.6	6.5	
		35.6	6.9		1.0		44.6	11.9	
**Spain**		29.0	13.0				40.6	17.4	
	18.8				12.5	6.2	6.2	6.2	
**United Kingdom**	38.0						57.0		
		46.4	5.3		3.6		41.1	3.6	
**United States**	76.6				9.3		11.7	1.4	
		65.4	8.0		4.3		9.7		
**Argentina**		50.0	25.0		8.3		16.7		
**Brazil**	87.5						12.5		
**Taiwan**		22.0	14.1		10.5		10.5		39.3
**Indonesia**		46.7	3.3	16.7			26.7	6.7	

The proportion of PWID was studied and reported in 49 articles and ranged from 0.6% to 100% [see Additional file A5]. Thirty-five out of 92 studies reported analyses on risk factors associated with HCV seroprevalence. In 24 of these studies, IVDU was present as a risk factor significantly associated with HCV seroprevalence. HCV Ab prevalence in anincarcerated setting appears to be increased when IDU prevalence is higher within that incarcerated setting [see Additional file A6]. HIV coinfection was reported in 36 sources and varied between 0% in Australia, Belgium, Egypt, Ethiopia, Switzerland, and one study in Missouri, USA, to 42.7% in a Spanish prison [see Additional file A5].

**Fig. 3:**
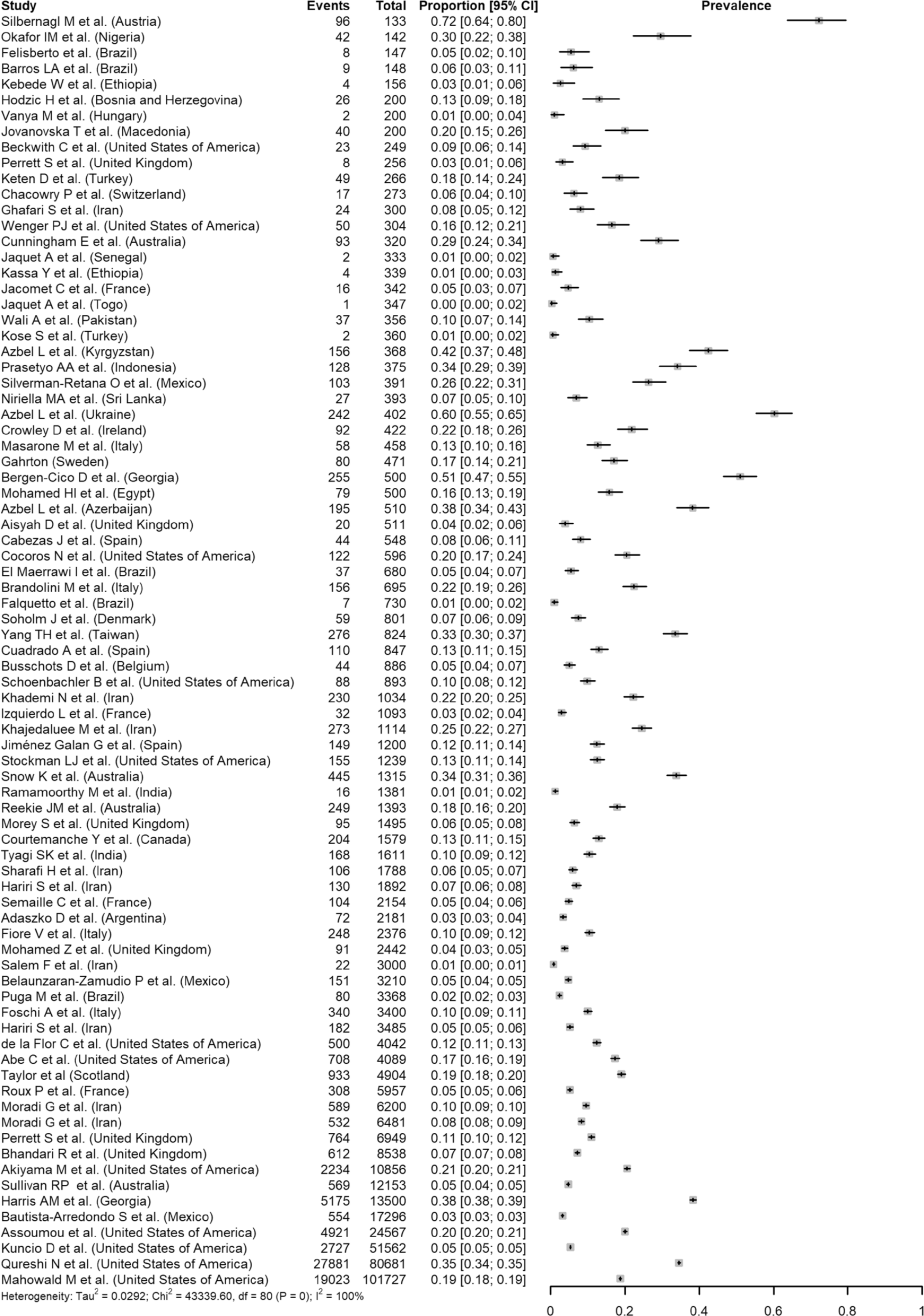
Results of the meta-analyses for HCV Ab prevalence in incarcerated settings

In the meta-analysis, regardless of which transformation was used, I^2^ was high, for example 99.8% (95%CI 99.8–99.8%) when using the double arcsine transformation. In the GLMM, I^2^ was still high (99.9%). Analyses excluding the smallest study (Silbernagl et al*.*, 2018), which had a very high prevalence estimate (72%; 95%CI 64–80%), did not affect the results. This indicates that almost all observed heterogeneity reflects true differences in estimates between studies, and a pooled prevalence estimate cannot be obtained (Fig. 3). Subgroup analyses revealed a significant difference regarding the study region (*p* = 0.004). Based on results from univariate analyses, study region (*p* = 0.022), proportion of PWID (*p* < 0.001), and proportion of males (*p* = 0.119), as well as their two-way interactions, were included in the multivariable meta-regression model. The final meta-regression model included significant interactions between region and proportion of PWID, as well as between proportion of PWID and proportion of males, and explained 76.0% of the between-study heterogeneity among the 42 studies reporting PWID that were included in this model.

**Fig. 4:**
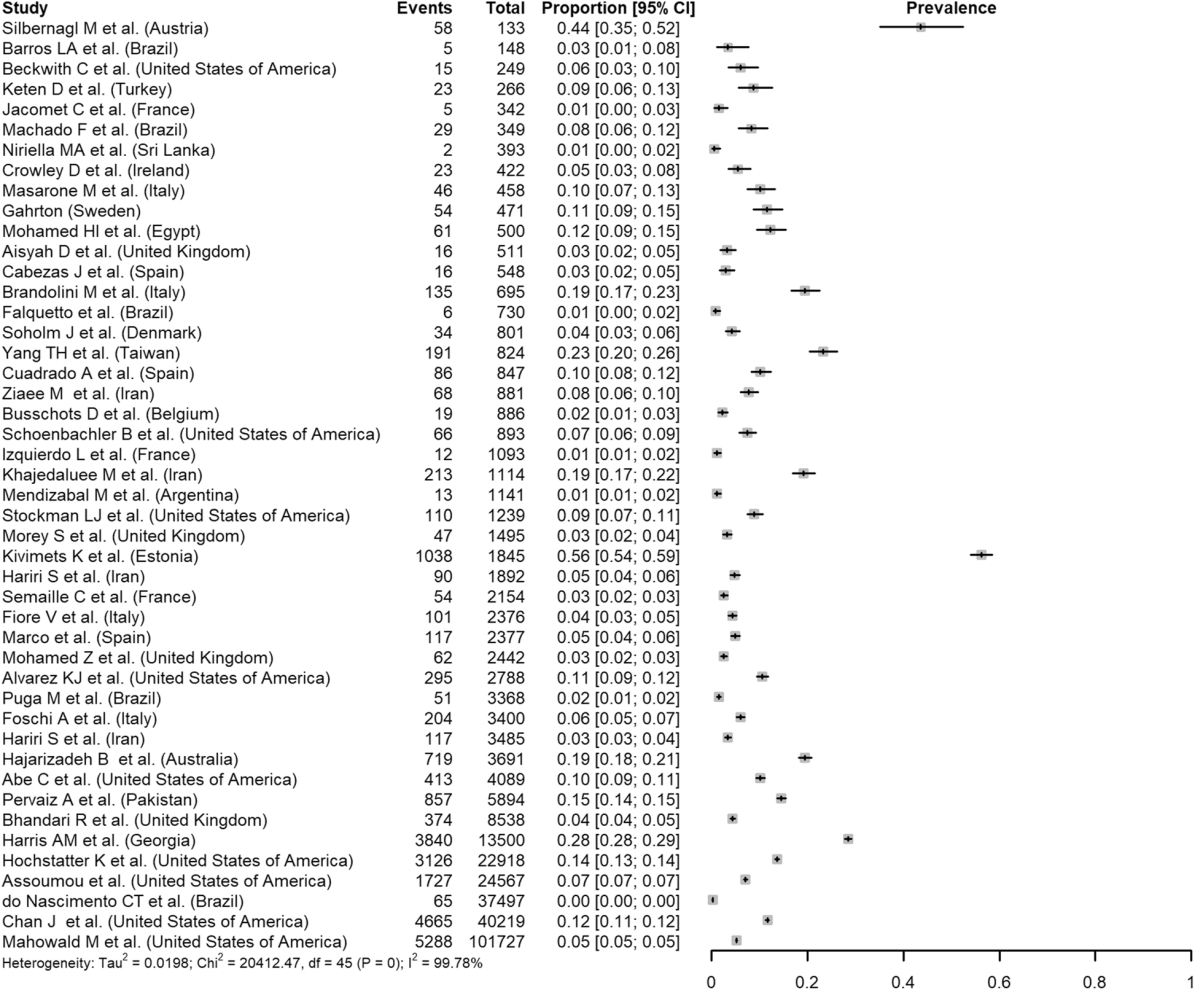
Results of the meta-analyses for HCV RNA prevalence in incarcerated settings

Regarding HCV RNA prevalence in inmates, 46 studies were included in a meta-analysis. I^2^ was 99.8% (95%CI 99.8–99.8%), indicating that almost all observed heterogeneity reflects true differences in prevalence between studies (Fig. 4). Subgroup analyses revealed a significant difference between regions (*p* < 0.001). Based on results from univariate analyses, study region (*p* = 0.002), single- vs multi-site study (*p *= 0.064), proportion HIV (*p *= 0.001), and proportion of PWID (*p *< 0.001) would be included in the multivariable meta-regression model. However, due to the limited number of studies reporting on HIV (*n* = 24) and PWID (*n* = 22) these were not included in the multivariable model. The final meta-regression model included only region (*p* = 0.002) and explained 67.1% of the between-study heterogeneity among studies reporting HCV RNA prevalence.

## Discussion

Gathering data on HCV prevalence in different settings is key to tracking the path to micro-elimination by 2030. Micro-elimination is a practical approach involving population segmentation to tailor concentrated elimination efforts to specific subgroups of the population. In addition, mapping the prevalence of HCV RNA is crucial to assess the need for treatment within incarcerated settings. This is the first meta-analysis to determine the HCV RNA prevalence in incarcerated settings worldwide. Despite the efforts, the data were too heterogeneous to establish a reliable pooled prevalence.

As expected, HCV Ab prevalence was higher than in the general population, identifying the population in an incarcerated setting as a risk group to target for micro-elimination. This elevated HCV prevalence could impose a financial burden on the health care system. It is striking that the prevalence of HCV Ab varies widely, even within a region of high-income countries such as Western Europe. The high numbers from Italy, Ireland, Scotland (the United Kingdom), and Sweden stand out. As the studies from Italy and Ireland are monocentric studies, they are not nationally representative. This is confirmed by the fact that the other Italian studies reported a substantially lower prevalence (10.0%-12.7% compared to 22.4%) [41, 42]. In this study, we also attempted to collect data on HCV RNA prevalence in incarcerated settings. However, only half of the studies reported these data, which is important for treatment and ultimately eliminating the virus.

It is crucial to obtain qualitatively strong and thus multicenter data on HCV prevalence that are representative of a country. Just under half of the studies were multicenter, and the quality assessment showed that half of all articles were rated A, B1, or B2.

Reporting HCV genotype is also essential as there are still countries where pangenotypic treatment is not yet available [114]. From the data collected on HCV genotypes, it appears that genotypes 1 and 3 are the most prevalent in an incarcerated setting. This finding supports the argument that HCV in individuals who are incarcerated is acquired primarily by IVDU since genotypes 1a and 3 are more prevalent in the PWID population worldwide than in the general population [115]. HCV/HIV coinfection varied between 0% and 42.7% and was highest in Italy, Estonia, Pakistan, and Spain. Dolan et al. associated higher HIV prevalence within an incarcerated setting with higher prevalence of PWID, but we lack sufficient data to support this argument.

Moreover, treatment for chronic HCV infection in closed settings can be delivered with sustained virologic response rates similar to those in community settings [116]. Treatment in closed settings would benefit individuals who are incarcerated and provide significant public health benefits, including reduced transmission and lowering the disease burden associated with chronic HCV infection. However, the high cost of treatment often remains a burden to implement it widely. After all, it places a heavy financial burden on the healthcare budgets of closed institutions [7].

This meta-analysis has several limitations and demonstrates the pitfalls of this kind of study. First, we only searched for articles published from 2013 onwards. Larney et al. concluded that many countries lacked epidemiological data on the extent of HCV infection in detained populations and that more efforts were needed. The available data were too heterogeneous and would have no added value in the current meta-analysis [7]. In addition, we did not include grey literature or unpublished results. Second, selection bias was a potential problem in some studies due to high non-response rates or flaws in the study design. Further, variables that could explain between-study heterogeneity, such as duration of incarceration, sexual behavior, and the number of previous incarcerations, were not analyzed because consistently reported data were mainly lacking. Thereby, even in the articles mentioning high-risk behavior in incarcerated settings such as IVDU, not everything may have been reported. Given that admitting particular risk behavior may have social implications for the individual who is incarcerated, self-reported data might not be reliable, leading to underreporting of high-risk behavior [115, 117]. As in the study of Larney et al., we could not include data from countries with a large population of incarcerated people, such as China or Russia. This study also did not include information on the incarcerated setting and incarceration policies in a country. These items could explain the wide variation in prevalence between different countries within a given region.

## Conclusions

To conclude, HCV in incarcerated settings is still a significant problem with a higher prevalence than in the general population. In addition, data on HCV RNA are scarce, while these data are critical for HCV micro-elimination within the an incarcerated setting. Therefore, more studies need to be conducted to estimate the actual global burden of HCV in incarcerated settings. Future studies should consistently report more information on study design, type and size of the incarcerated setting, specific high-risk population included (e.g., PWID and people living with HIV), and use a better study method (e.g., multicenter and/or multiple sample types) to ensure data quality. Finally, it is of utmost importance to start screening for HCV (Ab and RNA) in incarcerated settings to give clear, reliable and recent figures to plan further treatment. This is all in the context of meeting the 2030 WHO targets which are only less than a decade away.

## Supplementary Information

**Additional file 1. **A1. PRISMA 2020 Checklist.

**Additional file 2. **A2. Year of publication of included sources.

**Additional file 3. **A3. Classification system for assessment of study methodologies.

**Additional file 4. **A4. Country of origin of included sources.

**Additional file 5. **A5. Included prevalence sources with additional risk factors and the prevalence of HIV coinfection, people who inject drugs and male incarcerated individuals (*n*=92; 36 countries).

**Additional file 6. **A6. Dissemination of the prevalence hepatitis C antibodies and the prevalence of people who inject drugs (PWID) within the different studies.

## Data Availability

The authors confirm that the data supporting the findings of this study are available within the article and its supplementary materials.

## Abbreviations

HCV: Hepatitis C virus
WHO: World Health Organization
Ab: Antibody
GLMM: Generalized linear mixed model
IVDU: Intravenous drug use
OAT: Opioid agonist therapy
PWID: People who inject drugs
