# Supplementary material for: Hepatitis C prevalence in incarcerated settings between 2013–2021: a systematic review and meta-analysis
Source: BMC Public Health. 2022 Nov 24;22:2159. doi: 10.1186/s12889-022-14623-6 (PMC9685883; doi:10.1186/s12889-022-14623-6)
Supplement: Supplementary file 2 — Additional file 2. A2. Year of publication of included sources. [file 12889_2022_14623_MOESM2_ESM.docx]

**Additional file 2**

**A2. Year of publication of included sources**

Note: Data for 2021 are to August
