# Supplementary material for: Hepatitis C prevalence in incarcerated settings between 2013–2021: a systematic review and meta-analysis
Source: BMC Public Health. 2022 Nov 24;22:2159. doi: 10.1186/s12889-022-14623-6 (PMC9685883; doi:10.1186/s12889-022-14623-6)
Supplement: Supplementary file 3 — Additional file 3. A3. Classification system for assessment of study methodologies. [file 12889_2022_14623_MOESM3_ESM.docx]

**Additional file 3**

**A3. Classification system for assessment of study methodologies**

| Grade | Hepatitis C prevalence data |
| --- | --- |
| A | Multi-site seroprevalence study with >1 sample types |
| B B1 | Seroprevalence study, single sample type and multiple sites |
| B2 | Seroprevalence study, multiple sample types and a single site |
| C | Seroprevalence study, single sample type and single site |
| D | Registration or notification of cases of hepatitis infection |
| E | Prevalence study using self-reported hepatitis status, saliva or RNA testing only |
| U | Ungraded: Report with methodology unknown |
