# Supplementary material for: Hepatitis C prevalence in incarcerated settings between 2013–2021: a systematic review and meta-analysis
Source: BMC Public Health. 2022 Nov 24;22:2159. doi: 10.1186/s12889-022-14623-6 (PMC9685883; doi:10.1186/s12889-022-14623-6)
Supplement: Supplementary file 4 — Additional file 4. A4. Country of origin of included sources. [file 12889_2022_14623_MOESM4_ESM.docx]

**Additional file 4**

**A4. Country of origin of included sources**

| **Country** | **n** |
| --- | --- |
| Argentina | 2 |
| Australia | 5 |
| Austria | 1 |
| Azerbaijan | 1 |
| Belgium | 1 |
| Bosnia and Herzegovina | 1 |
| Brazil | 7 |
| Canada | 1 |
| Denmark | 1 |
| Egypt | 1 |
| Estonia | 1 |
| Ethiopia | 2 |
| France | 4 |
| Georgia | 2 |
| Hungary | 1 |
| India | 2 |
| Indonesia | 1 |
| Iran | 10 |
| Ireland | 1 |
| Italy | 4 |
| Kyrgyzstan | 1 |
| Macedonia | 1 |
| Mexico | 3 |
| Nigeria | 1 |
| Pakistan | 2 |
| Scotland | 1 |
| Senegal | 1* |
| Spain | 4 |
| Sri Lanka | 1 |
| Sweden | 1 |
| Switzerland | 1 |
| Taiwan | 1 |
| Togo | 1* |
| Turkey | 2 |
| Ukraine | 1 |
| United Kingdom | 6 |
| United States | 15 |
| **Total** | **92** |

*Same article
