# Supplementary material for: Hepatitis C prevalence in incarcerated settings between 2013–2021: a systematic review and meta-analysis
Source: BMC Public Health. 2022 Nov 24;22:2159. doi: 10.1186/s12889-022-14623-6 (PMC9685883; doi:10.1186/s12889-022-14623-6)
Supplement: Supplementary file 5 — Additional file 5. A5. Included prevalence sources with additional risk factors and the prevalence of HIV coinfection, people who inject drugs and male incarcerated individuals (n=92; 36 countries). [file 12889_2022_14623_MOESM5_ESM.docx]

**Additional file 5**

**A5. Included prevalence sources with additional risk factors and the prevalence of HIV coinfection, people who inject drugs and male incarcerated individuals (n=92; 36 countries)**

| **Ref. no** | **Grade** | **Country** | **Year pub.** | **Years data coll.** | **Cohort** | **Multi-site** | **N** | **N anti-HCV+** | **Anti-HCV prev. %** | **HCV RNA prev. %** | **Risk factors significantly associated to HCV** | **HIV coinfection (%)** | **PWID (%)** | **Male**  **(%)** |
| --- | --- | --- | --- | --- | --- | --- | --- | --- | --- | --- | --- | --- | --- | --- |
| **Eastern Europe (n=10; 8 countries)** | | | | | | | | | | | | | | |
| 23 | B1 | Azerbaijan | 2015 | 2014 | Prospective | Yes | 510 | 195 | 38.2 | ND | IVDU (AOR 12.9 95% CI 7.1-23.3), shared paraphernalia (AOR 4.8 95% CI 1.6-14.6) | ND | 31.9 | 90.6 |
| 24 | C | Bosnia and Herzegovina | 2017 | 2013 | Prospective | No | 200 | 26 | 13.0 | ND | DU before imprisonment, IVDU before imprisonment, tattooing in prison and having sex with homosexual partners | ND | 38.1 | 100 |
| 25 | E | Estonia | 2018 | 2014-2015 | Retrospective | No | 1,845 | ND | ND | 56.3 | ever DU (AOR 6.51 95% CI 5.12–8.28), HIV (AOR 2.56 95% CI 1.92–3.43), previous incarceration (AOR 3.16 95% CI 2.48–4.04) | 36.5 | ND | 94.4 |
| 26 | B2 | Georgia | 2017 | 2016 | Prospective | No | 500 | 255 | 60.0 | ND | ND | ND | ND | 96 |
| 27 | B1 | Georgia | 2019 | 2013-2015 | Retrospective | Yes | 13,500 | 5,175 | 38.0 | 28.4 | ND | ND | ND | ND |
| 28 | B1 | Hungary | 2017 | 2014 | Prospective | Yes | 200 | 2 | 1.0 | ND | ND | ND | ND | ND |
| 29 | B1 | Macedonia | 2014 | ND | Prospective | Yes | 200 | 40 | 20.0 | ND | ND | ND | ND | ND |
| 30 | C | Turkey | 2016 | 2014-2015 | Retrospective | No | 266 | 49 | 17.7 | 8.6 | ND | ND | 20.3 | 89.5 |
| 31 | B1 | Turkey | 2019 | ND | Prospective | Yes | 360 | 2 | 0.5 | ND | ND | ND | ND | ND |
| 32 | B1 | Ukraine | 2013 | 2011 | Prospective | Yes | 402 | 242 | 60.2 | ND | ND | ND | 48.7 | 79.9 |
| **Western Europe (n=25; 10 countries)** | | | | | | | | | | | | | | |
| 33 | A | Austria | 2018 | ND | Prospective | No | 133 | 96 | 74.4 | 45.0 | IVDU (OR 330.33 95% CI 25.9-4433.2), age first IVDU (OR 0.90 95% CI 0.8-1.0) | ND | 78.9 | 78.0 |
| 34 | B1 | Belgium | 2021 | 2019-2020 | Prospective | Yes | 886 | 44 | 5.0 | 2.1 | IVDU (AOR 24.6 95% CI (5.5-215.2), heroin past 6 months (AOR 24.6 95% CI 5.5-215.2) | 0 | 20.4 | 93.1 |
| 35 | B1 | Denmark | 2019 | 2016-2017 | Prospective | Yes | 801 | 59 | 7.4 | 4.2 | IVDU (AOR 182 95% CI 71.6-465), lifetime years in prison >10y (AOR 6.0 95% CI 2.2-16.5) | ND | 8.5 | 96.9 |
| 36 | B1 | France | 2013 | 2010 | Prospective | Yes | 2,154 | 104 | 4.8 | 2.5 | ND | 0.1 | ND | 74.6 |
| 37 | A | France | 2014 | 2004-2010 | Prospective | Yes | 5,957 | 308 | 5.2 | ND | IVDU without snorting (IRR 32.3 95% CI 20.1-51.8), IVDU with snorting (IRR 30.9 95% CI 21.3-45.0) , drug snorting alone (IRR 2.2 95% CI 1.4-3.5) , tattoos and/or piercing (IRR 1.2 95% CI 0.9-1.6), sharing toiletry items (IRR 1.4 95% CI 0.8-2.5) | ND | 6.7 | 93.0 |
| 38 | B1 | France | 2016 | 2012-2013 | Prospective | Yes | 342 | 16 | 4.7 | 1.5 | ND | ND | 7.7 | 94.1 |
| 39 | C | France | 2019 | 2017 | Retrospective | No | 1,093 | 32 | 2.9 | 1.1 | Older (44.8 95% CI 51.7-37.9) | 0.4 | ND | 89.8 |
| 40 | C | Ireland | 2019 | 2017 | Prospective | No | 422 | 92 | 22.8 | 5.5 | IVDU, having received a prison tattoo or a non-sterile community tattoo and sharing needle/syringe | 11.0 | 6.4 | 100 |
| 41 | C | Italy | 2013 | 2006-2008 | Prospective | No | 695 | 156 | 22.4 | 19.4 | HIV positive (89.6%; 95% CI: 79.7%-95.7%) | 38.4 | ND | 98.0 |
| 42 | B1 | Italy | 2016 | ND | Prospective | No | 3,400 | ND | 10.0 | 6.0 | ND | ND | ND | ND |
| 43 | C | Italy | 2020 | 2018-2019 | Prospective | No | 458 | 58 | 12.7 | 10.0 | ND | 1.5 | 18.7 | 94.0 |
| 44 | B1 | Italy | 2021 | 2019 | Prospective | Yes | 2,376 | 248 | 10.4 | 4.3 | ND | 3.0 | 23.0 | 98.0 |
| 45 | D | Spain | 2014 | 1992-2011 | Retrospective | No | 2,377 | ND | ND | 4.9 | IVDU (OAR 7.3 95% CI 4.8-11.0), HIV (OAR 2.0 95% CI 1.1-3.4) | 1.9 | 7.1 | ND |
| 46 | C | Spain | 2018 | 2016-2017 | Prospective | No | 847 | 110 | 13.0 | 10.2 | ND | 15.9 | 7.1 | 100 |
| 47 | C | Spain | 2019 | 2015 | Retrospective | No | 1,200 | 149 | 12.4 | ND | ND | 42.7 | ND | ND |
| 48 | C | Spain | 2021 | 2019-2021 | Prospective | No | 548 | 44 | 8.0 | 2.9 | ND | 12.5 | 2.0 | 86.5 |
| 49 | B1 | United Kingdom | 2013 | 2010-2011 | Prospective | Yes | 4,904 | 933 | 19.0 | ND | ND | ND | 32.0 | 95.0 |
| 50 | A | United Kingdom | 2018 | 2011-2013 | Prospective | Yes | 511 | 20 | 4.0 | 3.1 | ND | 1.0 | ND | ND |
| 51 | B2 | United Kingdom | 2019 | 2016-2017 | Prospective | No | 1,495 | 95 | 6.4 | 3.1 | ND | 0.1 | ND | ND |
| 52 | E | United Kingdom | 2019 | 2016 | Prospective | No | 256 | 8 | 3.1 | ND | ND | ND | ND | 100 |
| 53 | B1 | United Kingdom | 2020 | 2016-2020 | Prospective | Yes | 8,538 | 612 | 7.2 | 4.4 | ND | ND | ND | ND |
| 54 | C | United Kingdom | 2020 | 2019-2019 | Prospective | No | 2,442 | 91 | 3.7 | 2.6 | ND | 3.3 | ND | ND |
| 55 | B1 | United Kingdom | 2020 | 2015-2017 | Retrospective | Yes | 6,949 | 764 | 11.0 | ND | ND | ND | ND | 100 |
| 57 | B1 | United Kingdom | 2019 | 2017 | Prospective | Yes | 471 | 80 | 17.0 | 11.5 | ND | ND | ND | 93.4 |
| 57 | E | Switzerland | 2018 | 2011-2013 | Prospective | No | 273 | 17 | 6.2 | ND | IVDU | 0 | 5.9 | 100 |
| **North America (n=16; 2 countries)** | | | | | | | | | | | | | | |
| 58 | B1 | Canada | 2018 | 2014-2015 | Prospective | Yes | 1,565 | 204 | 12.9 | ND | ND | ND | 21.2 | 84.4 |
| 59 | E | United States | 2013 | 2009-2013 | Prospective | Yes | 2,788 | ND | ND | 10.1 | IVDU (AOR 64.8 95% CI 37.4-112.1), sex partners who were IVDU (AOR 8.0, 95% CI 3.9-16.2), HIV (AOR 4.3 95% CI 2.2-8.3) | 15.9 | 8.9 | 54.4 |
| 60 | C | United States | 2014 | 2009-2011 | Prospective | No | 596 | 122 | 20.5 | ND | ND | ND | 49.0 | 85.7 |
| 61 | C | United States | 2014 | 2012-2013 | Prospective | No | 304 | 50 | 16.4 | ND | age (OR 1.1 95% CI 1.04-1.15), IVDU (OR  53.9 95% CI 17.8-163.2) | 0 | 25.3 | 81.9 |
| 62 | C | United States | 2015 | 2011-2012 | Prospective | No | 51,562 | 2,727 | 3.0 | ND | ND | ND | ND | 86.0 |
| 63 | B1 | United States | 2016 | 2012-2014 | Prospective | Yes | 249 | 23 | 9.2 | ND | Past and/or recent IVDU | ND | 17.7 | 90.6 |
| 64 | B1 | United States | 2016 | 2004-2012 | Prospective | Yes | 101,272 | 19,023 | 18.7 | 5.2 | ND | ND | ND | 80.9 |
| 65 | D | United States | 2016 | 2012-2014 | Prospective | Yes | 893 | 88 | 13.2 | 7.4 | ND | 27.7 | 18.2 | 86.0 |
| 66 | B1 | United States | 2016 | 2014-2015 | Prospective | Yes | 1,239 | 155 | 12.5 | 8.9 | ND | ND | 16.0 | 85.2 |
| 67 | C | United States | 2017 | 2013-2014 | Retrospective | No | 10,856 | 2,234 | 20.6 | ND | IVDU (AOR 35.0 95% CI 28.5-43.0), Women (AOR 1.3 95% CI 1.1-1.5), Hispanic (AOR 2.1 95% CI 1.8-2.4), non-Hispanic white (AOR 1.7 95% CI 1.5-2.1) | 14.8 | 17.7 | 93.6 |
| 68 | C | United States | 2017 | 2015-2016 | Prospective | No | 4,042 | 500 | 16.4 | ND | ND | ND | ND | ND |
| 69 | E | United States | 2017 | 2015 | Retrospective | No | 22,918 | ND | ND | 13.6 | ND | 5.0 | ND | ND |
| 70 | E | United States | 2019 | 2012-2016 | Retrospective | No | 24,567 | 4,921 | 20.0 | 7.0 | ND | ND | ND | ND |
| 71 | C | United States | 2020 | 2017 | Retrospective | No | 4,089 | 708 | 17.3 | 10.1 | ND | ND | ND | 77.5 |
| 72 | D | United States | 2020 | 2014-2017 | Retrospective | No | 40,219 | ND | ND | 11.6 | ND | ND | ND | 88.8 |
| 73 | D | United States | 2021 | 2000-2019 | Retrospective | No | 80,681 | 27,881 | 34.6 | ND | IVDU (AOR 34.9 95% CI 24.6-49.5), 1946-1955 (AOR 13.0 95% CI 11.9-14.2), men (AOR 1.4 95% CI 1.3-1.5), Hispanic (AOR 4.2 95% CI 3.9-4.4), non-Hispanic (AOR 3.8 95% CI 3.5-4.0) | 2.2 | 16.4 | 75.0 |
| **Latin America (n=12; 3 countries)** | | | | | | | | | | | | | | |
| 74 | B1 | Argentina | 2018 | 2015-2017 | Prospective | Yes | 2,181 | 72 | 3.3 | ND | ND | ND | ND | 89.0 |
| 75 | B1 | Argentina | 2020 | 2018-2020 | Retrospective | Yes | 1,141 | ND | ND | 1.1 | Age (OR 1.07 95% CI 1.0-1.1), IVDU (OR 12.66 95% CI 3.3-48.5) | 0.2 | 1.7 | 73.5 |
| 76 | C | Brazil | 2013 | 2007-2008 | Prospective | No | 148 | 9 | 6.1 | 3.4 | IVDU (OR 18.8 95% CI 6.7-52.8), Length of incarceration (OR 4.1 95% CI 0.9-19.3) | ND | ND | 0 |
| 77 | A | Brazil | 2013 | 2010 | Prospective | Yes | 730 | 7 | 1.0 | 0.8 | ND | ND | ND | ND |
| 78 | C | Brazil | 2015 | 2007 | Prospective | No | 680 | 37 | 5.3 | ND | IVDU (OR 4.4 95% CI 1.5-7.4), STI (OR 2.3 95% CI 1.2-4.4), age >30y (OR 1.9 95% CI 1.2-3.0) | ND | 5.9 | 100 |
| 79 | B1 | Brazil | 2017 | 2013-2014 | Prospective | Yes | 3,368 | 80 | 2.4 | 1.5 | IVDU past year (AOR 5.13 95% CI 2.0-13.4), age 41-50 (AOR 11.0 95% CI (4.6-26.7) | 6.3 | 1.0 | 84.6 |
| 80 | C | Brazil | 2019 | 2015 | Prospective | No | 147 | 8 | 5.4 | ND | ND | ND | 4.8 | ND |
| 81 | D | Brazil | 2019 | 2016-2017 | Retrospective | No | 349 | ND | ND | 8.3 | ND | 0.3 | ND | 86.0 |
| 82 | D | Brazil | 2020 | 2017-2018 | Retrospective | Yes | 37,497 | ND | ND | 0.2 | ND | ND | ND | ND |
| 83 | B1 | Mexico | 2015 | 2010 | Prospective | Yes | 17,296 | 554 | 3.2 | ND | ND | ND | ND | 91.3 |
| 84 | B1 | Mexico | 2017 | 2011-2012 | Prospective | Yes | 3,210 | 151 | 4.8 | ND | ND | ND | 9.2 | ND |
| 85 | B1 | Mexico | 2017 | 2010-2013 | Prospective | Yes | 391 | 103 | 3.3 | ND | IVDU (OR 43.1 95% CI 33.0-53.7) | 1.4 | ND | ND |
| **Central Asia (n=1; 1 country)** | | | | | | | | | | | | | | |
| 86 | B1 | Kyrgyzstan | 2016 | 2014 | Prospective | Yes | 368 | 156 | 49.7 | ND | ND | 20.4 | 30.4 | 78.0 |
| **East Asia (n=1; 1 country)** | | | | | | | | | | | | | | |
| 87 | C | Taiwan | 2020 | 2019 | Prospective | No | 824 | 276 | 33.5 | 23.2 | ND | 8.9 | ND | ND |
| **South Asia (n=6; 4 countries)** | | | | | | | | | | | | | | |
| 88 | C | India | 2016 | 2015 | Prospective | No | 1,381 | 16 | 1.2 | ND | ND | ND | 20.0 | 91.1 |
| 89 | C | India | 2018 | 2016 | Prospective | No | 1,611 | 168 | 10.4 | ND | IVDU | ND | ND | ND |
| 90 | B1 | Indonesia | 2013 | 2009 | Prospective | Yes | 375 | 128 | 34.1 | ND | IVDU (OR 2.5, 95% CI 1.528-3.989) | 4.0 | 77.7 | 77.1 |
| 91 | E | Pakistan | 2015 | 2007-2009 | Prospective | No | 5,894 | ND | ND | 14.6 | convicted | ND | ND | ND |
| 92 | C | Pakistan | 2019 | 2017 | Prospective | No | 356 | 37 | 10.4 | ND | ND | 32.4 | ND | 98.6 |
| 93 | B1 | Sri Lanka | 2015 | ND | Prospective | Yes | 393 | 27 | 6.9 | 0.5 | ND | ND | 4.3 | 82 |
| **West Asia (n=10; 1 country)** | | | | | | | | | | | | | | |
| 94 | B1 | Iran | 2013 | 2008-2009 | Prospective | Yes | 3,000 | 22 | 0.7 | ND | ND | 13.6 | ND | 100 |
| 95 | B1 | Iran | 2014 | 2009-2010 | Prospective | Yes | 881 | ND | ND | 7.7 | ND | 1.5 | 4.4 | 82.1 |
| 96 | B1 | Iran | 2016 | 2008 | Prospective | Yes | 1,114 | 273 | 24.5 | 19.1 | ND | ND | ND | 89.0 |
| 97 | B1 | Iran | 2018 | 2015 | Prospective | Yes | 6,200 | 589 | 9.5 | ND | history of DU (AOR 4.08 95% CI 2.56–6.27), age >30 (AOR 2.68 95% CI 2.01–3.56), and tattoos (AOR 1.67 95% CI: 1.35–2.07). | ND | 17.0 | 96.5 |
| 98 | C | Iran | 2019 | 2016 | Prospective | No | 300 | 24 | 8.0 | ND | IVDU (OR 4.1 95% CI 1.6-10.2) | ND | 18.8 | 100 |
| 99 | B1 | Iran | 2019 | ND | Prospective | Yes | 1,034 | 230 | 22.2 | ND | ND | ND | ND | 100 |
| 100 | B1 | Iran | 2019 | 2016 | Prospective | Yes | 6,481 | 532 | 8.2 | ND | history of DU (AOR 5.8 95% CI 3.5 - 9.4), age >30 (AOR 5.0, 95% CI 3.7 - 6.9), history of tattooing (AOR 2.4 95% CI 2.0 - 3.0), history of imprisonment (AOR 2.2 95%CI 1.8 - 2.8), being single (AOR 1.9 95% CI 1.5 - 2.4), low education (AOR 1.8 95%CI 1.0 - 3.3), history of piercing (AOR 1.3 95% CI 1.0 - 1.5). | ND | 15.0 | 97.3 |
| 101 | C | Iran | 2019 | 2017-2018 | Prospective | No | 1,788 | 106 | 5.9 | ND | ND | ND | ND | 100 |
| 102 | C | Iran | 2020 | 2017-2018 | Retrospective | No | 3,485 | 182 | 5.2 | 3.4 | ND | ND | ND | 100 |
| 103 | C | Iran | 2020 | 2018 | Prospective | No | 1,892 | 130 | 6.9 | 4.8 | ND | ND | 13.0 | 96.4 |
| **Australasia (n=5; 1 country)** | | | | | | | | | | | | | | |
| 104 | B1 | Australia | 2014 | 2004-2010 | Prospective | Yes | 1,393 | 249 | 29.8 | ND | ever IVDU (IRR 16.3 95% CI 9.5-27.9), IVDU last month (IRR 19.9 95% CI 11.6-33.9), imprisonment (IRR 1.67 95% CI 1.2-2.3) | 0.1 | 58.3 | 90.0 |
| 105 | B1 | Australia | 2017 | 2005-2014 | Prospective | Yes | 320 | 93 | 29.1 | ND | Frequency injecting (HR 3.08 (95% Ci 1.7-5.7) | ND | 100 | 72.0 |
| 106 | D | Australia | 2017 | 2008-2010 | Prospective | Yes | 1,315 | 445 | 33.8 | ND | ND | ND | 58.9 | 78.9 |
| 107 | B1 | Australia | 2021 | 2018 | Prospective | Yes | 3,691 | ND | ND | 19.0 | IVDU past 6 monthes (AHR 6.14 95% CI 3.2-11.9), Age at enrolment (AHR 0.92 95% CI 0.9-1.0) | 0 | 21.0 | 82.0 |
| 108 | D | Australia | 2021 | 2003-2017 | Prospective | Yes | 1,315 | 445 | 33.8 | ND | ND | ND | ND | ND |
| **West Africa (n=3; 2 countries)** | | | | | | | | | | | | | | |
| 109 | C | Senegal | 2016 | 2014 | Prospective | No | 333 | 2 | 0.6 | ND | ND | ND | 0.6 | 100 |
| 109 | C | Togo | 2016 | 2013 | Prospective | No | 347 | 1 | 0.3 | ND | ND | ND | 0.6 | 100 |
| 110 | C | Nigeria | 2020 | 2018 | Prospective | No | 142 | 42 | 29.6 | ND | ND | ND | 19.0 | 90.8 |
| **Sub-Saharan Africa (n=2; 1 countries)** | | | | | | | | | | | | | | |
| 111 | C | Ethiopia | 2017 | 2016 | Prospective | No | 156 | 4 | 2.6 | ND | history of blood transfusion (AOR 5.7 95% CI 5.2–6.2) | 0 | ND | 93.0 |
| 112 | C | Ethiopia | 2021 | 2020 | Prospective | No | 339 | 4 | 1.2 | ND | history of blood transfusion (AOR 27.6 95% CI 2.3–325.6), history of dental extraction (AOR 39.9 95% CI 3.4-68.6) | ND | 3.8 | 89.4 |
| **Middle East and North Africa (n=1; 1 countries)** | | | | | | | | | | | | | | |
| 113 | C | Egypt | 2013 | ND | Prospective | No | 500 | 79 | 15.8 | 12.2 | IVDU (AOR 4.1 95% CI 2.8–5.3), incarceration >10 years (AOR 3.4 95% CI 1.2–4.8), shared toiletries (AOR 3.9 95% CI 2.5–5.2), diabetes mellitus (AOR 3.9 95% CI 3.2–5.9), dental procedures (AOR 4.7 95% CI 2.7–6.3), tattooing (AOR 2.8 95% CI 2.2–4.7) | 0 | 8.4 | 78.2 |

Abbreviations: ND, not described. CI, confidence interval; DU, drug use; IVDU, intravenous drug use, PWID; people who inject drugs, AOR; adjusted odds ratio, OR; odds ratio, IRR; incidence rate ratio, AHR; adjusted hazard ratio, HR; hazard ratio. Notes: No studies identified for countries of North Asia, Pacific Islands or Caribbean
