# Supplementary material for: Hepatitis C prevalence in incarcerated settings between 2013–2021: a systematic review and meta-analysis
Source: BMC Public Health. 2022 Nov 24;22:2159. doi: 10.1186/s12889-022-14623-6 (PMC9685883; doi:10.1186/s12889-022-14623-6)
Supplement: Supplementary file 6 — Additional file 6. A6. Dissemination of the prevalence hepatitis C antibodies and the prevalence of people who inject drugs (PWID) within the different studies. [file 12889_2022_14623_MOESM6_ESM.docx]

**Additional file 6**

**A6. Dissemination of the prevalence hepatitis C antibodies and the prevalence of people who inject drugs (PWID) within the different studies. It can be inferred that, in general, a higher prevalence of PWID is associated with a higher HCV Ab prevalence**
